# Stricture of the Urethra and Enlargement of the Prostate

**Published:** 1902-09

**Authors:** 


**Stricture of the Urethra and Enlargement of the Prostate.** By P. J. Freyer. Second Edition. Pp. vii., 132. London: Baillière, Tindall & Cox. 1902.

We reviewed the first edition of this book, and observe that our comments have received notice. The alterations in the present edition only affect portions dealing with affections of the prostate. With regard to prostatectomy, he states that later experience has induced him to modify very materially many of the views put forward in the first edition. Though he classes McGill's operation among the "partial" ones in contradistinction to his claim to "total" prostatectomy, yet he states that in performing the former he has removed both lateral lobes projecting into the bladder, the whole weighing 3 ounces, the patient gaining complete control over micturition. In a few of his cases of the McGill operation, he says expulsive power was not regained "owing to the existence of complete atony of the muscles of the bladder." But, later on, when advocating his claim to the originality of "total" extirpation, he states, with regard to atony of the bladder, "that no such condition exists, even in the most advanced instances of the disease."

We owe much to Mr. Freyer for his writings upon the anatomy and surgery of the prostate, and chiefly the fact that by aid of the finger in the rectum the prostate may be removed as a whole instead of by *morcellement*, although we fail to see that by the latter the operation need be any the less total in its completion.

